# Heme-oxygenase-1 implications in cell morphology and the adhesive behavior of prostate cancer cells

**DOI:** 10.18632/oncotarget.1826

**Published:** 2014-03-16

**Authors:** Geraldine Gueron, Jimena Giudice, Pia Valacco, Alejandra Paez, Belen Elguero, Martin Toscani, Felipe Jaworski, Federico Coluccio Leskow, Javier Cotignola, Marcelo Marti, Maria Binaghi, Nora Navone, Elba Vazquez

**Affiliations:** ^1^ Department of Biological Chemistry, School of Sciences, University of Buenos Aires, IQUIBICEN-CONICET, Intendente Guiraldes 2160, CABA; ^2^ Department of Pathology and Immunology, Baylor College of Medicine, One Baylor Plaza, Houston, TX, USA; ^3^ CEQUIBIEM-Department of Biological Chemistry, School of Sciences, University of Buenos Aires, IQUIBICEN-CONICET; ^4^ Department of Genitourinary Medical Oncology, The University of Texas, M. D. Anderson Cancer Center, Houston, TX, USA

**Keywords:** Prostate Cancer, Heme-oxygenase-1, Muskelin, E-cadherin, B-catenin, Adherens Junctions

## Abstract

Prostate cancer (PCa) is the second leading cause of cancer death in men. Although previous studies in PCa have focused on cell adherens junctions (AJs), key players in metastasis, they have left the molecular mechanisms unexplored. Inflammation and the involvement of reactive oxygen species (ROS) are critical in the regulation of cell adhesion and the integrity of the epithelium. Heme oxygenase-1 (HO-1) counteracts oxidative and inflammatory damage. Here, we investigated whether HO-1 is implicated in the adhesive and morphological properties of tumor cells. Genes differentially regulated by HO-1 were enriched for cell motility and adhesion biological processes. HO-1 induction, increased E-cadherin and β-catenin levels. Immunofluorescence analyses showed a striking remodeling of E-cadherin/β-catenin based AJs under HO-1 modulation. Interestingly, the enhanced levels of E-cadherin and β-catenin coincided with a markedly change in cell morphology. To further our analysis we sought to identify HO-1 binding proteins that might participate in the regulation of cell morphology. A proteomics approach identified Muskelin, as a novel HO-1 partner, strongly implicated in cell morphology regulation. These results define a novel role for HO-1 in modulating the architecture of cell-cell interactions, favoring a less aggressive phenotype and further supporting its anti-tumoral function in PCa.

## INTRODUCTION

Prostate cancer (PCa) is the most frequently diagnosed cancer in American men as well as the second leading cause of cancer death [1]. Incidence increases with patient age and represents the most important risk factor. Localized PCa can be cured in most cases, but when the disease escapes the confines of the gland, the prospects for cure decrease drastically.

Cellular adhesion involves cell-to-extracellular matrix (ECM) and cell-cell interactions, and is a primary feature of the architecture of tissues. A disturbance in epithelial cell adhesion leads to a more invasive phenotype, a hallmark of tumor progression [2]. Certain cell adhesion molecules (CAMs) play a pivotal role in the development of recurrent and distant metastasis [3]. A strong correlation between the loss of CAMs expression and a higher grade in prostate carcinoma has been reported [3]. The main CAMs at adherens junctions (AJs) in epithelial cells are E-cadherin and β-catenin [4, 5]. E-cadherin is a membrane glycoprotein [6] involved in the assembly of epithelial cells [2] and its loss is associated with increased cellular invasion and the subsequent metastasis [2, 7-9]. E-cadherin–binding proteins, including β-catenin, transduce adhesion-elicited signals to the cell interior and provide a linkage to the actin cytoskeleton. The intracellular trafficking and mobilization of AJ components at the cell surface turn out to be paramount in several malignancies [10]. Hence the molecular mechanisms underlying the regulation of CAMs rise as an important hurdle to overcome in aggressive PCa.

Inflammation has been defined as an enabling characteristic for its contributions to the well-known cancer hallmark capabilities [2]. The inflammatory tumor microenvironment is a fertile niche that releases ROS, which accelerates the malignant transformation and appears as a fine tuner of the adhesive behavior of cells [11, 12]. Elevated endogenous ROS generation was found in several tumors and has been associated also with the regulation of angiogenesis [13, 14] through VEGF expression [15]. ROS modulate the molecular crosstalk between cell-matrix and cell-cell adhesion receptors [16], stabilizing or destabilizing AJs mediated by distinct cadherins, including E-, N-, and VE-cadherin. The mechanisms for such modulation include either biochemical modifications of CAMs, epigenetic alterations of the cadherin promoter, or regulation of small GTPases modulting cadherin-dependent cell-cell adhesion [16].

The induction of heme oxygenase 1 (HO-1), the rate-limiting enzyme in heme degradation, represents an essential event in cellular responses to pro-oxidative and pro-inflammatory insults [17], maintaining the cellular homeostasis [18]. Carbon monoxide (CO) and bilirubin from heme oxygenase-1 suppress ROS generation [19].

HO-1 has been proposed to act as a cellular biosensor. Previous reports from our laboratory documented the inhibition of ROS production by HO-1 in PCa cells [13]. Moreover, we also confirmed that pharmacological or genetic modulation of HO-1 induces its nuclear localization and inhibits cell proliferation, migration and invasion [20]. It also impairs tumor growth and angiogenesis *in vivo* and down-regulates the expression of target genes associated with inflammation [13, 20]. However, the implication of HO-1 in the adhesive capability of cells needs yet to be addressed.

This study aimed to gain insights into the functional significance of HO-1 expression in the epithelial architecture, in the cell shape and its adhesive properties. We demonstrate that HO-1 is implicated in the modulation of cellular adhesion in PCa, up-regulating E-cadherin and β-catenin expression, favoring these proteins relocation to the cell membrane. Furthermore, through a proteomics approach we identified a novel interaction between HO-1 and Muskelin, a mediator of cell spreading and cytoskeletal responses. Overall, these results support an unprecedented regulatory mechanism of HO-1 over the maintenance of the epithelial cell morphology and architecture.

## RESULTS

### HO-1 induction promotes down-regulation of genes associated with cell locomotion and chemotaxis

**FIGURE 1:**
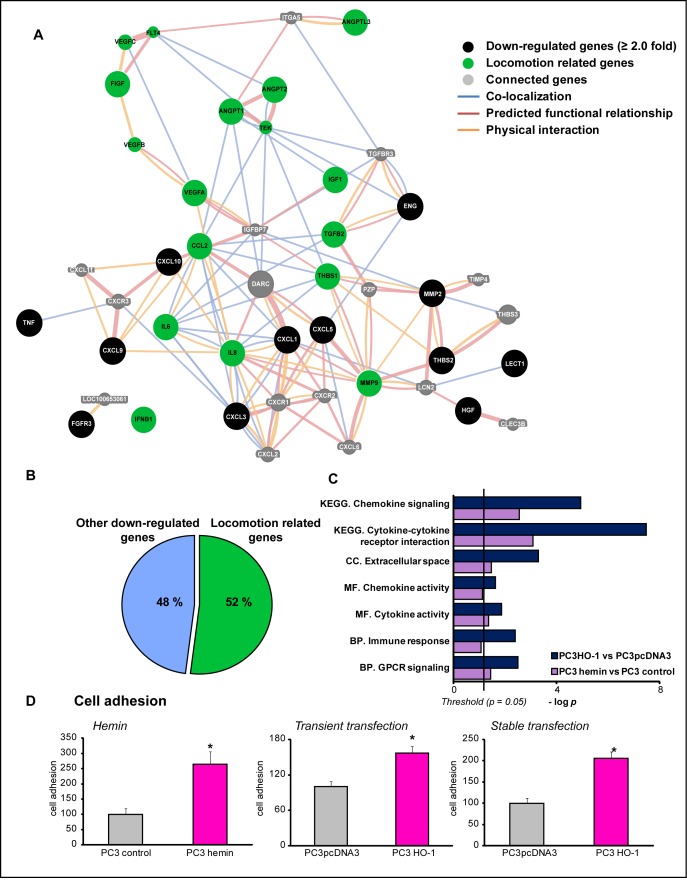
HO-1 regulated genes are involved in cell locomotion and adhesion A) GeneMANIA software was used to perform the interaction network between HO-1 down-regulated (≥2.0 fold) genes in PCa cell lines: hemin (70 μM, 24 h) treated PC3 cells *vs*. control cells, and PC3HO-1 *vs* PC3pcDNA3. Black circles represent down-regulated genes, green circles show locomotion related genes, and connected genes are in grey circles. Lines between circles are as follow: blue represent co-localization interactions, red lines predicted functional relationship based on literature, and orange lines, physical interactions. B) HO-1 down-regulated genes were classified into locomotion associated genes and others. C) Differentially expressed genes in hemin-treated PC3 cells *vs* controls (purple bars) and PC3HO-1 *vs* PC3pcDNA3 cell lines (blue bars) were assigned to different GO ontologies: biological processes (BP), molecular functions (MF), cellular components (CC) and KEGG pathways (KEGG). D) Hemin treated PC3 cells, PC3 transiently or stably transfected with pcDNA3HO-1 (PC3HO-1) and respective controls were assayed for cellular adhesion to collagen. One representative from at least three independent experiments is shown. Results are shown as mean ± s.e.m (*P<0.05).

We have previously reported that PCa cells over-expressing HO-1 as well as PCa cell lines with high HO-1 endogenous levels displayed repressed levels of MMP9 [20], a metalloproteinase highly correlated with PCa invasion and metastasis [21]. Microarray analysis also revealed that HO-1 down-regulated the expression of other several pro-inflammatory and angiogenic genes. Here we used GeneMANIA [22] and DAVID database [23] to extend our query on other genes, related biological pathways and gene ontology (GO) categories [24]. Our input gene set included those genes up- or down-regulated by HO-1, either pharmacologically (hemin treatment, a potent inducer of HO-1) or genetically (PC3 cells over-expressing HO-1, PC3HO-1). The results showcased a gene network where 52% of the genes were associated with cell locomotion and motility (Fig. 1A, B). This gene network is interconnected either by reported gene co-localization, predicted functional relationship or physical interaction. Enrichment ontology analysis of the data sets from PC3 cells treated with hemin and PC3HO-1 compared to their respective controls, allows identification of gene groups associated with a particular physiologic or pathologic molecular or cellular function. We found a statistically significant and consistent association with categories including: chemokine signaling and cytokine-cytokine receptor interaction (KEGG pathways), extracellular space (GO-cellular component), chemokine and cytokine activity (GO-molecular function), immune response and GPCR (G protein coupled receptor) signaling (GO-biological process) (Fig. 1C and Supplemental Table 1). Moreover, among the network of related GO terms associated with biological process we found: migration and proliferation, locomotory behavior and chemotaxis regulation (Fig. 1C, Supplemental Table 1 & 2). We also performed an enrichment analysis using Metacore software, on the data sets corresponding to genes modulated in the PC3HO-1 versus (*vs*) control cell line. Results rendered a significant association of the HO-1 regulated genes with several proteins located in the extracellular space and cell membrane for the prostatic neoplasm disorder ontology (Supplemental Fig. 1). In summary, all the bioinformatics analysis showcased a strong association to the extracellular space, a compartment highly correlated with the adhesive behavior of cells.

### HO-1 over-expression increases cellular adhesion in PCa cell lines

To investigate whether this strong association of HO-1 with genes implicated in the extracellular space had an impact on PCa cellular adhesion, we evaluated PC3 cell line (androgen-insensitive) capability to adhere to collagen-coated plates. Pharmacological induction of HO-1 by hemin, revealed a significant increase of adherent cells (2.6 fold, *P*<0.05*;* Fig. 1D). Moreover, HO-1 over-expressing PC3 cells also showed a significant increase in cellular adhesion (Fig. 1D) compared to control cell lines. This was observed for both, HO-1 transiently and stably transfected cells (1.5 and 2.0 fold respectively, *P*<0.05*;* Fig. 1D), which demonstrates that HO-1 is capable of modulating the adhesive response of PCa cells and it is consistent with our previous published work where cells displaying high levels of HO-1 appear to acquire a less malignant phenotype, with decreased cellular proliferation, migration and invasion [20].

### Up-regulation of E-cadherin and β-catenin by forced-expression of HO-1 in PCa cells and prostate tumor xenografts

**FIGURE 2:**
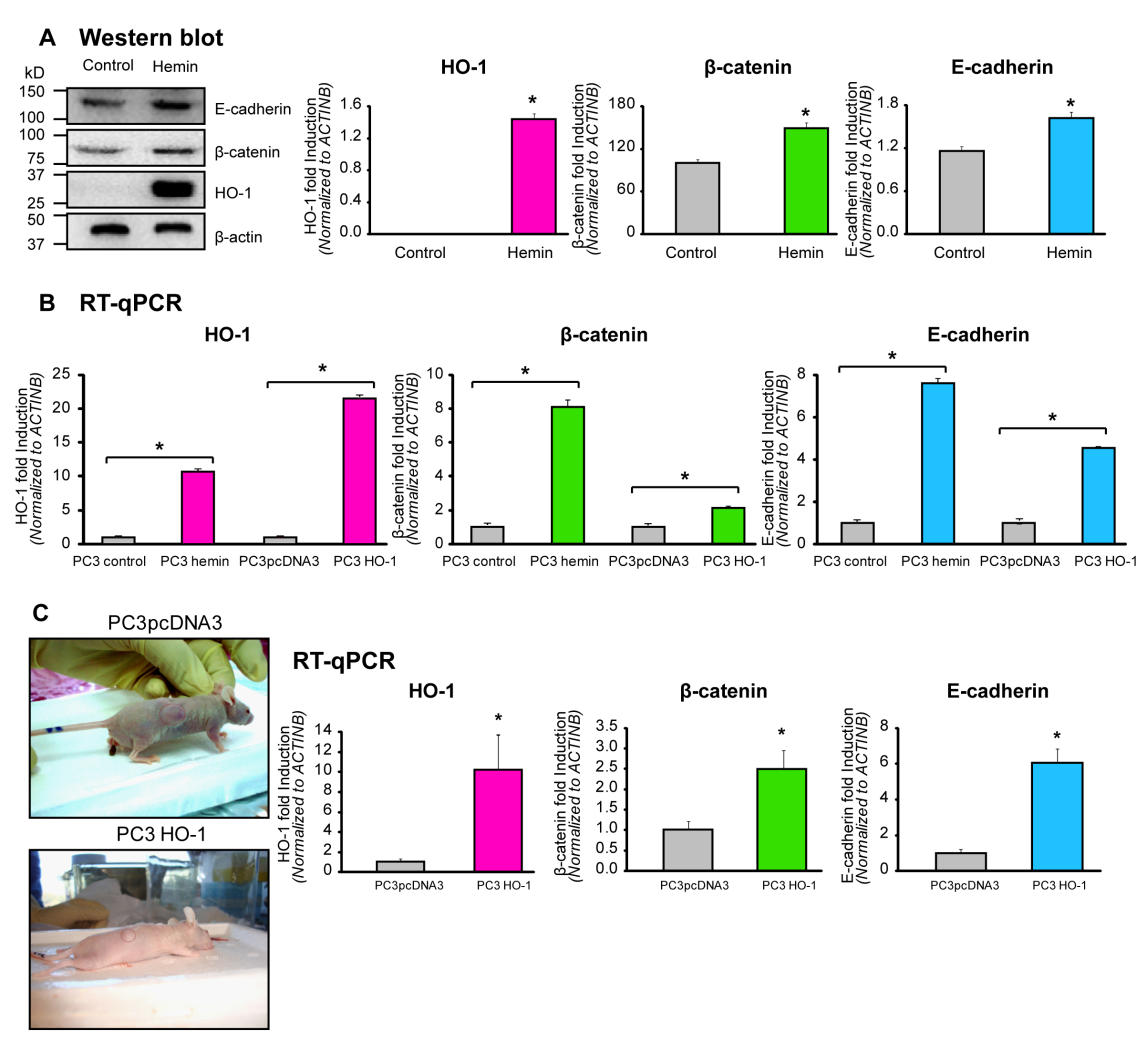
HO-1 upregulates the expression of E-cadherin and β-catenin in PCa cells and prostate tumor xenografts A) PC3 cells were cultured for 24 h and then exposed to hemin (70 μM, 24 h) or vehicle (control). Total protein was extracted, and HO-1, E-cadherin and β-catenin expression was analyzed by Western blotting (WB). β-actin levels are shown as loading control. Quantification was performed by densitometry analysis using Image J software. Protein band intensities were normalized to β-actin. The results are expressed as mean ± s.e.m (*P<0.05). B) mRNA expression levels of HO-1, E-cadherin and β-catenin were analyzed by Real Time-PCR (RT-qPCR) in PC3 cells transfected with pcDNA3HO-1 or empty vector or exposed to hemin (70μM, 24h) or vehicle. Data were normalized to β-actin. One representative from at least three independent experiments is shown (*P<0.05). C) Athymic nude (*nu/nu*) mice were injected *s.c.* in the right flank with PC3 cells overexpressing HO-1 (PC3HO-1) or control (PC3pcDNA3). Animals were sacrificed after 23 days (left upper and lower panels) and tumors were excised. Tumors were harvested, snap-frozen in liquid nitrogen and processed for RNA isolation. HO-1, E-cadherin and β-catenin mRNA levels were analyzed by RT-qPCR. Data were normalized to β-actin. One representative from at least three independent experiments is shown (*P<0.05).

The intracellular trafficking and mobilization of AJ components at the cell surface are important for growth and development. Therefore, we evaluated whether HO-1 was also capable of modulating key CAMs and interfering with cell-cell interactions. Our results show that treatment of PC3 cells with hemin resulted in greatly enhanced E-cadherin and β-catenin expression at the protein and mRNA levels (Fig. 2A, B). These epithelial markers showed also significantly induced mRNA levels in the PC3HO-1 cell line (4.5 fold induction and 2.1 fold induction for E-cadherin and β-catenin, respectively, *P*<0.05; Fig. 2B). To determine the role of HO-1 *in vivo*, we injected *s.c.* 3.5×10^6^ PC3HO-1 or PC3pcDNA3 cells in the right flank of athymic nude male mice [20]. Accordingly, PC3HO-1 xenografts displayed higher mRNA expression levels of E-cadherin and β-catenin (6.0-fold and 2.5 fold induction, respectively, *P*<0.05; Fig. 2C). We have previously published that these tumors were smaller in size and displayed reduced MMP9 expression and activity levels [20]. Altogether, these results confirm that HO-1 modulates these CAMs *in vitro* and *in vivo*, supporting its implication in cellular adhesion.

### Re-arrangement of E-cadherin and β-catenin-based adherens junctions by HO-1 up-regulation in PCa cells

**FIGURE 3:**
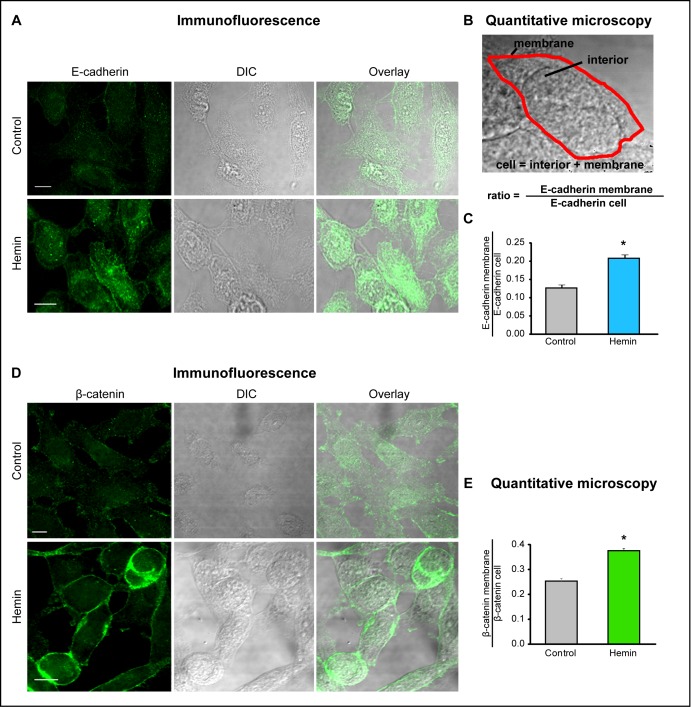
HO-1 promotes E-cadherin and β-catenin re-distribution to the cell membrane in PCa cells A) E-cadherin expression and cellular distribution was visualized by immunofluorescence staining of PC3 cells treated with hemin (70 μM, 24 h) or vehicle (control). A representative image for each group is shown. E-cadherin localization displayed a significant increase in cell-cell border staining in cells treated with hemin compared to a more diffuse pattern observed in control cells (scale bar: 10 μm). B) Segmentation of plasma membrane (red) and cell interior was performed. Intensity of E-cadherin in the membrane region and the whole cell was measured and the ratio E-cadherin membrane/E-cadherin cell was calculated. C) Quantitative microscopy indicating the augmented levels of the E-cadherin membrane/E-cadherin cell ratio when PC3 cells were exposed to hemin (*P<0.05). D) β-catenin expression and cellular distribution visualized by immunofluorescence staining of PC3 cells treated with hemin or vehicle. A representative image for each group is shown. β-catenin localization also showed a significant increase in cell-cell border staining after hemin treatment compared to control cells (scale bar: 10 μm). E) β-catenin membrane/β-catenin cell ratio was calculated as in D (*P<0.05).

**FIGURE 4:**
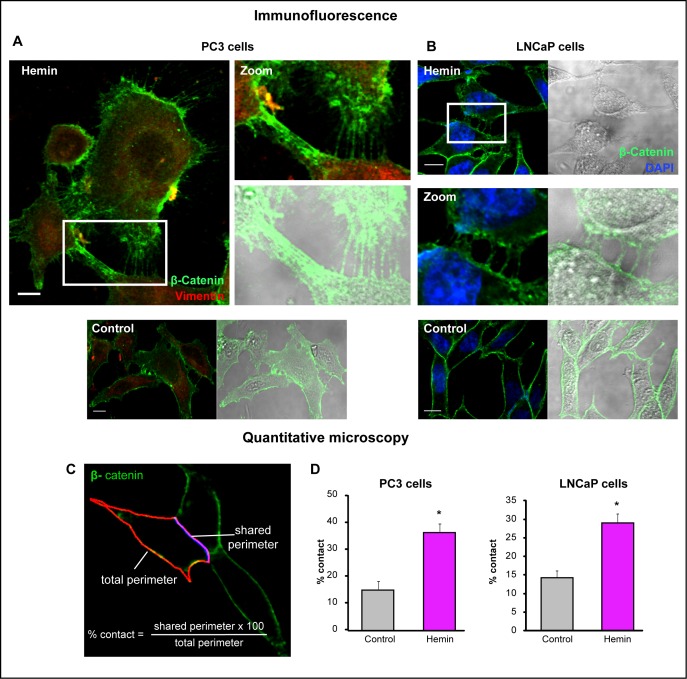
Forced-expression of HO-1 re-arranges the β-catenin-based adherens junctions in PCa cells A) and B) β-catenin expression was visualized by immunofluorescence in LNCaP (A) or PC3 (B) cells treated with hemin (70 μM, 24 h) or vehicle (control). Representative images for each cell line are shown. β-catenin localization is observed at cell-cell adherens junctions revealing a network of filopodia from neighboring cells under hemin treatment. DAPI (blue) was used for nuclear staining and vimentin (red) for cytoplasmic staining (scale bars: 10 μm). White open boxes represent the zoomed images. C) Segmentation of the cell perimeter (red) and the shared perimeter (violet) was performed and the percentage of contact (shared perimeter x100/total perimeter) was calculated. D) Quantitative microscopy of the percentage of contact when PC3 and LNCaP cells were exposed to hemin (*P<0.05).

The stable E-cadherin-based AJs play a pivotal role in the integrity of the epithelium and the maintenance of tissue homeostasis. To better understand the nature of alterations of cell-cell interactions during neoplastic evolution, we examined the expression patterns of E-cadherin and β-catenin under HO-1 up-regulation in PCa cells. Immunofluorescence staining, not only confirmed the augmented levels of these epithelial markers under forced-expression of HO-1, but also revealed a striking rearrangement of their localization pattern towards the cell membrane (Fig. 3A, D). Moreover, the quantitative image analysis of the ratio between E-cadherin at the cell membrane and total E-cadherin (Fig. 3B) indicated a significant increase in the percentage of cell-cell contact among tumoral cells under HO-1 overexpression (Fig. 3C, E) compared to controls. In order to examine these cell-cell contact regions with high fluorescent activity in closer detail, we explored β-catenin-based AJs in PC3 cells at a higher magnification. We also extended our observations to LNCaP cells (androgen-sensitive). Interestingly, imaging (Fig. 4A, B) revealed a network of filopodia from neighboring cells that have established contact. We then evaluated whether the augmented AJs and the observed cell-cell connecting filopodia were associated with an increase in the percentage of contact between cells. We analyzed the shared perimeter between cells (Fig. 4C) exposed to hemin. In both cell lines, HO-1 induction correlated with a significant increase in the percentage of contact among cells (Fig. 4D).

**FIGURE 5:**
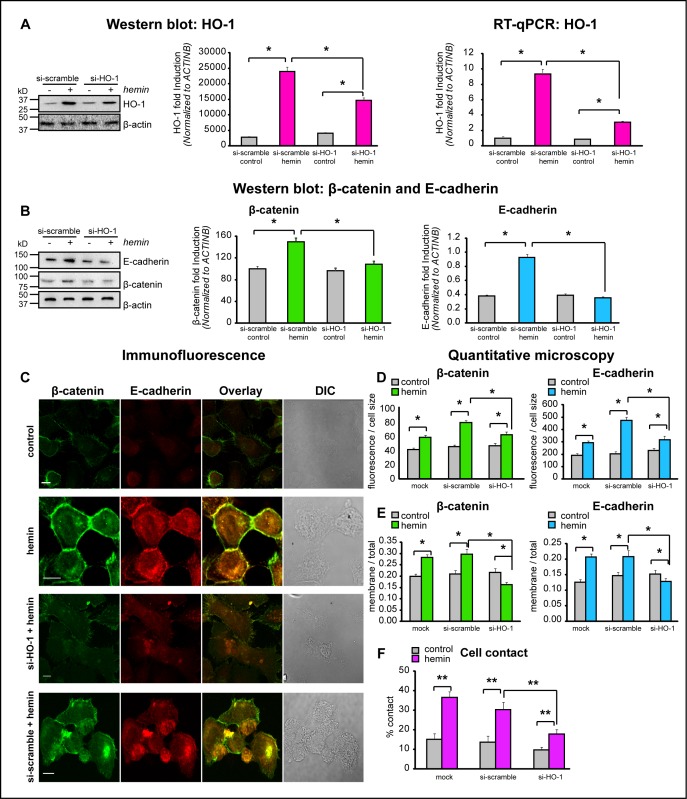
HO-1 interference by siRNA directly affects the expression and cellular distribution of E-cadherin and β-catenin in PCa cells A) PC3 cells were exposed to hemin (70 μM, 24 h) or vehicle (control) and transfected with siRNAHO-1 (si-HO-1) or scrambled siRNA (si-scramble). Total protein and mRNA were extracted and HO-1 expression levels were analyzed by Western Blotting (WB) and RT-qPCR. β-actin was used as loading control. Protein quantification was performed by densitometry analysis using Image J software and band intensities were normalized to β-actin. mRNA HO-1 levels were normalized to β-actin. One representative from at least 3 independent experiments is shown (*P<0.05). B) Cells were treated as in A) and E-cadherin and β-catenin protein levels were analyzed by WB. β-Actin was used as loading control. Quantification was performed as in A) (*P<0.05). C) Immunofluorescence analysis of E-cadherin and β-catenin in PC3 cells treated as described in A). Cells were fixed, stained with anti- E-cadherin and anti- β-catenin primary antibodies and secondary antibodies conjugated with Alexa-Fluor-488 (green) or Alexa-Fluor-555 (red). Cells were imaged by confocal microscopy using the same parameters for all the treatments. D) The florescence intensity for E-cadherin and β-catenin was calculated using Matlab and normalized to the cell size (*P<0.05; *n*≥10 cells for each condition). E) Segmentation of plasma membrane and cell interior was performed. E-cadherin and β-catenin intensity in both regions was measured and the ratios E-cadherin membrane/E-cadherin total and β-catenin membrane/β-catenin total were calculated (*P<0.05). F) The percentage of contact (shared perimeter x100/total perimeter) was calculated in individual cells (*P<0.01; *n*≥15 cells for each condition).

To confirm that HO-1 affects the expression and cellular distribution of E-cadherin and β-catenin in PCa cells, our next step was to knock down HO-1 *in vitro* using HO-1 small interfering RNA (siRNAHO-1). Hemin induced levels of HO-1 at the protein and mRNA levels were significantly decreased (39% and 67%, respectively, *P*<0.05; Fig. 5A) by siRNAHO-1. Accordingly, the induced levels of E-cadherin and β-catenin under hemin treatment were impaired when HO-1 was silenced in PC3 cells (Fig. 5B). The confocal microscopy imaging confirmed these findings. Cells were double stained for E-cadherin and β-catenin, showing not only enhanced fluorescence levels for both proteins under forced-expression of HO-1 (Fig. 5C, D), but also increased levels of these CAMs at the cell border with an augmented percentage of cell-cell contact (Fig 5E, F). Consistently with previous findings, the siRNAHO-1 reduced the hemin-induced expression and the membrane translocation of these CAMs and, in turn, the percentage of cellular contact (Fig5 C-F). Taken together, our data show that these primary AJ proteins (E-cadherin and β-catenin) are finely regulated by HO-1 expression in PCa cells, suggesting that HO-1 in these cells can modulate the architecture of cell-cell interactions.

### Structural analysis of PC3 cells under forced-expression of HO-1

**FIGURE 6:**
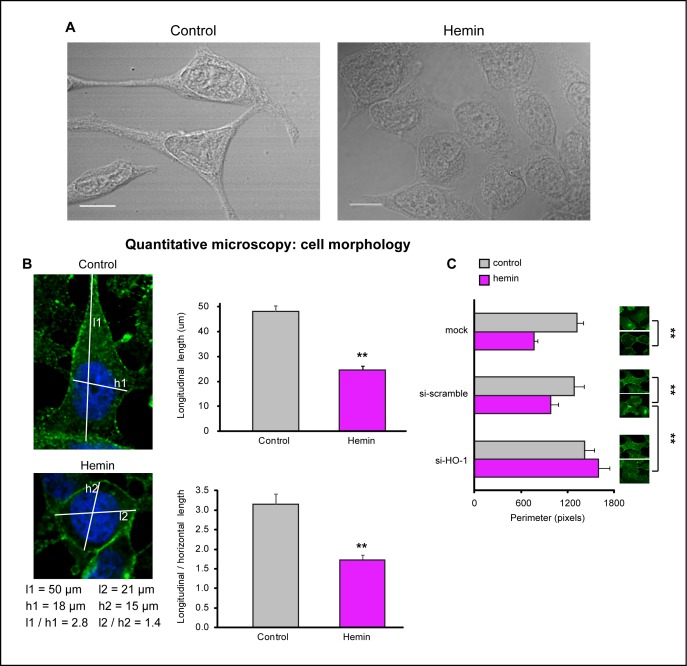
HO-1 is implicated in cell morphology regulation A) Cell morphology was examined by bright field microscopy in PC3 cells exposed to hemin (70 μM, 24 h) or vehicle (control). B) Longitudinal lengths and longitudinal length/horizontal length ratios were measured using ImageJ software (**P<0.01; *n*≥13 cells for each condition). C) Cell perimeter was analyzed with Matlab in PC3 cells exposed to hemin or vehicle and transfected with siRNAHO-1 (si-HO-1) or scrambled siRNA (si-scramble) (**P<0.01; *n*≥10 cells for each condition).

Intriguingly, the enhanced levels of E-cadherin and β-catenin in PCa cells under forced-expression of HO-1 described above (Fig. 4, 5) coincided with a markedly different appearance compared to control cells (Fig. 6A). To evaluate such morphological differences, bright field microscopy images were obtained. While untreated cells exhibited a more fibroblast-like morphology with long, loose cell to cell contact, branching cytoplasmic protrusions; hemin-treated cells showed a round/cuboidal, cobblestone epithelial morphology; with tight cell associations (Fig. 6A). Morphological cell measurements (longitudinal lengths and longitudinal length / horizontal length) verified the described qualitative observations. Hemin-treated cells displayed shorter cellular lengths compared to control cells (Fig. 6B) and a significant reduction in total cell perimeter compared to control cells *(P<0.01;* Fig. 6C). The addition of siRNAHO-1 restored the cellular perimeter (*P<0.01;* Fig. 6C). These results define a novel role for HO-1 in the modulation of cellular morphology, favoring an epithelial-like phenotype.

### Muskelin: a novel molecular partner of HO-1 implicated in cell morphology regulation

**FIGURE 7:**
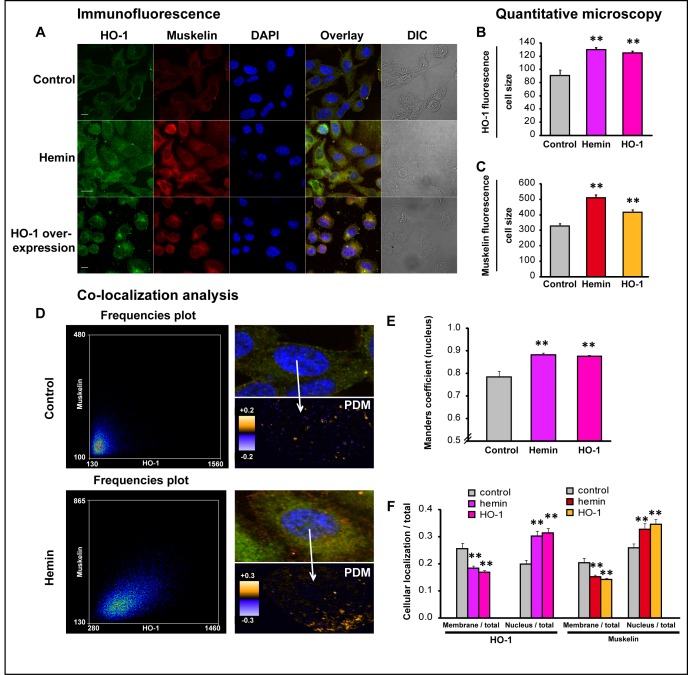
HO-1 increases muskelin expression and nuclear co-localization A) PC3 cells treated with hemin (70μM, 24 h) or vehicle (control) and PC3 cells over-expressing HO-1 display augmented levels of Muskelin compared to controls. Cells were fixed, stained with anti-HO-1 and anti-muskelin primary antibodies and secondary antibodies conjugated with Alexa-Fluor-488 or Alexa-Fluor-555. Cells were imaged by confocal microscopy applying the same parameters for all the experimental conditions. Image processing was performed similarly in all the treatments. B-C) The florescence intensity for HO-1 (B) and Muskelin (C) was measured and normalized to the cell size using Matlab (**P<0.01; *n*≥15 cells for each condition). D) Representative PDM graphs and frequency scatter plots were performed with the Image J intensity correlation analysis plug-in (x-axis: HO-1-Alexa-488, y-axis: Muskelin-Alexa-555). E) Manders coefficients in the nuclei of individual cells were calculated using Matlab (**P<0.01; *n*≥15 cells for each condition). F) PC3 cells treated with hemin or vehicle and PC3 cells over-expressing HO-1 were assayed by immunofluorescence. HO-1 and Muskelin fluorescence were measured in the plasma membrane region and in the whole cell, and the ratios membrane/total and nucleus /total were measured for each case (**P<0.01; *n*≥15 cells for each condition).

Evidence suggests that HO-1 may have a regulatory role beyond its enzymatic activity [20]. When translocating to the nuclei of PCa cells, it might need to partner up with other transcription factors or co-regulator of transcription to exert its regulatory function, given that it does not contain DNA binding motifs. To further our analysis we sought to identify HO-1 binding proteins that might participate in the regulation of cell morphology. For this purpose we screened for HO-1 interacting proteins using a proteomics approach. We performed Glutathione S-transferase (GST) pull-down assays using lysates from PC3 cells transiently transfected with GSTHO-1 or empty GST vector and further treated with interleukin-6 (IL-6). IL-6 is known to be a principal mediator of inflammation frequently elevated in patients with prostate carcinoma [25]. IL-6 treatment sets the scene of an inflammatory condition present in PCa, allowing us to screen for more possible HO-1 interactors, given that HO-1 conteracts oxidative and inflammatory damage. Our results show that HO-1 appears to interact with Muskelin (Supplemental Fig. 2), a transport factor that accompanies cargo delivery across different cytoskeletal transport systems [26, 27], and a nucleocytoplasmic mediator of cellular morphology and adhesiveness [28, 29]. Up-regulation of Muskelin under HO-1 induction in PCa cells was confirmed by confocal microscopy (P*<0.05; Fig. 7A-C and Supplemental Fig. 3). A high degree of nuclear overlay between HO-1 and Muskelin signals was observed when cells were exposed to hemin or genetically manipulated to over-express HO-1, compared to controls (Fig. 7 D). These observations were quantified by calculation of Manders coefficients (*P<0.01*; *n*=17 cells, Fig. 7 D, E). We plotted the product of the differences from the mean (PDM) showing the co-localization distribution and frequency scatter-plots. Signals close to a line at 45° confirmed the high co-localization degree (Fig. 7D). Interestingly, HO-1 and Muskelin exhibit similar sub-cellular dynamics. After HO-1 induction, both proteins seem to relocate from the cell membrane, towards the cell nuclei (Fig. 7F). Altogether, we have shown for the first time that HO-1 binds and up-regulates Muskelin, a specific factor involved in shaping cellular morphology and adhesive properties.

## DISCUSSION

While organ confined cancers have a high survival rate, metastatic cancers are associated with the vast majority of all cancer related deaths [1]. According to the 2013 Cancer Facts and Figures, there is a reported 100% five-year survival rate for PCa that is confined to the gland, while this five-year survival rate drops dramatically to 28% for PCa that has spread to a distant site. Such metastatic behavior relies partly on the loss of the tissue architecture, closely associated with cell-cell and to cell-ECM interactions [30]. When these associations are disorganized and cells display deficiencies of junctional structures, they are free to colonize other homing organs [31]. Because CAMs are major players in the intercellular adhesion of cancer cells [32-34], there has been increasing interest in the mechanisms that mediate the mis-regulation of their expression and localization patterns during the metastatic behavior of cells.

Inflammation can contribute to multiple hallmark capabilities by supplying bioactive molecules to the tumor microenvironment, including growth factors that sustain proliferative signaling, survival factors that limit cell death, pro-angiogenic factors, extracellular matrix-modifying enzymes that facilitate invasion, metastasis, and inductive hallmark-facilitating programs [11, 35, 36]. This inflammatory scenario creates a reactive microenvironment that fosters detachment, migration and invasion of malignant cells. In this context, we previously documented the critical role of HO-1, an anti-inflammatory and anti-oxidant protein in impairing tumor growth, inhibiting cell invasion and migration in PCa [13, 20, 37, 38]. The role of HO-1 in cancer still remains a controversial subject. Although some authors propose that HO-1 can be considered a “friend” aiding healthy tissues from the initiation of some types of cancers, when the disease starts to develop, HO-1 becomes a “false friend” [18]. Reports include pro-tumoral effects of HO-1 in lymphosarcoma, hepatoma, glioblastoma, melanoma, Kaposi sarcoma, squamous carcinoma and brain tumors [18, 39]. Alternatively, HO-1 appears to counteract tumor growth in non-small cell lung carcinoma [40] and in breast cancer it suppresses the invasive capacity of cells via MMP9 down-regulation [41]. Interestingly, a very recent work by Wegiel [42] showed a detailed analysis of a large cohort of PCa patients, confirming an enzymatically inactive form of HO-1 in the nuclei of cells and therefore a possible key regulator of cancer progression. They also exhibited that CO produced by HO-1, rapidly enhanced mitochondria activity of cancer cells, fueling cancer cell bioenergetics, resulting in metabolic exhaustion and tumor regression. Furthermore, CO also increased the sensitivity of tumor cells to chemotherapeutics while simultaneously preserving normal cell growth. Accordingly, in pancreatic cancer, Vitek [43] reported on the antiproliferative effects of CO and/or CORM-2 (a ruthenium-based, lipid-soluble carbon monoxide releasing molecule) pointing to the potential chemoadjuvant/chemotherapeutic use of carbon monoxide in pancreatic cancer.

It becomes increasingly clear that in addition to blocking cell proliferation, invasion and migration in PCa, other mechanisms including a direct effect on the adhesive behavior of cells could account for HO-1's anti-tumoral role in this disease. The bioinformatics analysis presented here rendered a gene network modulated by HO-1, closely associated with cell locomotion and motility. Moreover, the gene data sets from PC3 cells treated with hemin or the PC3HO-1 cell line *vs* their respective controls, had a strong association to the extracellular space; a compartment tightly linked to the adhesive behavior of cells and the development and progression of cancer [44-47]. We therefore analyzed the adhesive properties of PC3 cells under pharmacologic or genetic modulation of HO-1. Our results showed that HO-1 was capable of increasing cellular adhesion (Fig.1).

Epithelial cells communicate with their neighbor cell at AJs, through the extension of either lamellipodia (web-like branched actin network) or filopodia (linear bundles), coupling to the intracellular cytoskeleton, triggering specific signaling pathways and, in turn, contributing to cell migration and proliferation [5, 10, 48, 49]. E-cadherin plays a major role in the intercellular adhesion of cells, co-assembling with α-catenin and β-catenin, in a complex referred to as a “puncta” at sites of cell-cell contact [50]. The role of E-cadherin as a tumor suppressor is supported by the frequently observed loss of this molecule in a variety of invasive cancers, including breast [51], ovarian [8], pancreatic [52], gastric [53] and prostate cancer [54, 55]. In the latter case, cells with low expression of E-cadherin are more invasive [56]. Therefore, the absence of E-cadherin expression predicts the potential of metastasis to the bone [3, 57]. This evidence raises the question of how E-cadherin based AJs are regulated in the carcinogenic process.

Recent growing evidence highlights an important role of ROS in intra and inter-cellular signaling of CAMs raising the possibility that ROS constitute master regulators of the crosstalk between these fundamental cell adhesion proteins [16]. Strategies aimed at controlling ROS homeostasis to preserve the coordinated adhesive and signaling functions of CAMs might harbor important therapeutic potential for the pathology of cancer. Modulating HO-1 levels, could be a possible strategy, since it regulates ROS levels in cells [58, 59]. To elucidate the involvement of HO-1 in PCa AJs we analyzed the expression and cell localization patterns of E-cadherin and β-catenin. Our data show that HO-1 pharmacologic or genetic modulation induces both, E-cadherin and β-catenin *in vitro* and *in vivo* (Fig. 2). Moreover, it impacts on the localization of these CAMs towards the cell membrane (Fig. 3), which in turn, increases significantly the percentage of contact among cells (Fig. 4, 5). Our data is in line with our previous studies showing a reduced migratory ability of PCa cells under HO-1 modulation [20] and with other reports detailing how E-cadherin-mediated cell-cell adhesions prevent cell migration [32]. It is worth mentioning that we have previously documented that HO-1 down-regulates NF-κB [13], a major transcription factor involved in metastasis to bone, which is constitutively active in PCa [13]. Moreover, suppression of NF-κB, activity in PCa cells, stabilizes AJs [60]. In breast cancer, NF-κB also suppresses E-cadherin levels by inducing the expression of the master regulators Snail, Slug, ZIB1, ZIB2 and Twist [61]. Interestingly, the increased expression of NF-κB and Snail observed in gastric cancer tissues, coincides with a considerable reduction in E-cadherin levels in the cancer tissues compared to the normal gastric mucosa [62].

The reduction of ROS levels together with an impaired NF-κB signaling axis in PCa produced by HO-1 might ameliorate the inflammatory tumoral microenvironment, preserving the epithelial architecture. Insight into the molecular mechanisms and the nature of the signals involved in the regulation of CAMs and AJs remain to be fully elucidated. However, the experimental evidence and observations discussed in this paper, display the potential of HO-1 to interfere with these cellular processes and impact in the pathogenesis of PCa.

Interestingly, the enhanced levels of E-cadherin and β-catenin in PCa cells under HO-1 induction coincided with a markedly difference in cellular morphology (Fig. 6). PC3 cells under hemin treatment presented cobblestone appearance, forming clusters with a more round morphology and tight seals compared to control cells, which displayed long cytoplasmic protrusions, forming loose associations with adjacent cells with few opposing connecting filopodia. These changes were also visible in the PC3HO-1 cell line compared to its control cell line [20]. Cell structural differences have been reported in multiple types of cancers in connection to the levels of E-cadherin [51, 63]. However, the signaling pathways intervening in this modulation are yet to be clarified. To direct our analysis into potential molecular partners by which HO-1 could be altering these cellular changes we used a proteomics approach, with an eye toward proteins that could intimately be related to cellular morphology. We report here a novel interaction between HO-1 and Muskelin, an intracellular mediator of cellular morphology and adhesiveness [28], which exhibited similar sub-cellular dynamics to HO-1, relocating from the cell membrane towards the cell nuclei under hemin treatment (Fig. 7). In agreement with previously reported effects of Muskelin [27], the up-regulation of this protein is associated with a significant increase in cell attachment and with the restoration of cell morphology. While Muskelin–depleted cells display reduced attachment to fibronectin and altered spreading behavior, with significantly enlarged cell perimeters caused by increased ruffling and protrusive edges, cells with restored levels of Muskelin display normal morphology [28]. This phenotype was also confirmed in Muskelin siRNA-treated C2C12 cell line, which had similar specific increases in cell perimeter length and protrusions in fibronectin-adherent cells. These results strongly support our findings where HO-1 increases cell adhesion and restores cell morphology, possibly through the up-regulation of Muskelin, thus linking this molecule to the adhesive behavior of cells and to the regulation of their morphology. It will be of future interest to establish the functions of nuclear Muskelin in connection with HO-1. Noteworthy, Muskelin forms a complex with RANBP9/RANBPM, involved in transmembrane signaling [28] and a reported co-activator for the androgen receptor [64]. Coincidently, RANBPM also relocates to the cell nuclei of PC3 cells when HO-1 is induced (Supplemental Fig. 4). Altogether, the presence of Muskelin and HO-1 in the nucleus is suggestive of a regulatory rather than a structural role in PCa. Further studies will be needed to unravel the nuclear action of HO-1; but altogether, our data strongly support a key anti-tumoral role of HO-1 in prostate carcinogenesis ascertaining it as a logical target for therapeutic intervention.

## METHODS

### Cell culture, treatments, reagents and antibodies

LNCaP and PC3 cells were obtained from the American Type Culture Collection (Manassas, VA) and were routinely cultured in RPMI 1640 (Invitrogen) supplemented with 10% fetal bovine serum (FBS). PC3 stable transfected cells (PC3HO-1 and PC3pcDNA3) were previously described [20].

Hemin was obtained from SIGMA-Aldrich (UK). For treatments, cells were incubated 24 h in RPMI media containing 10% FBS and then were exposed to hemin (70 μM, 24 h), unless stated otherwise.

Polyclonal and monoclonal anti-HO-1 antibodies were from Stressgen Biotechnologies Corp. (San Diego, CA). Monoclonal anti-E-cadherin and anti-β-catenin antibodies were from Cell Signaling, Technology (Beverly, MA). Anti-β-Actin antibody was purchased from Sigma (UK). Anti-mouse and anti-rabbit secondary antibodies conjugated with HRP were from Amersham Ltd (UK). Secondary antibodies conjugated with Alexa Fluor 488 or Alexa Fluor 555 were from Molecular Probes, Invitrogen (Grand Island, NY)

### Plasmids and transient transfections

The human pcDNA3-HO-1 expression vector was kindly provided by Dr. M. Mayhofer (Clinical Institute for Medical and Chemical Laboratory Diagnostics, University of Vienna, Austria).

PC3 cells were plated in 12-well (1.2×10^5^cells per well). Expression vectors (2 μg) were transfected using Lipofectamine 2000 from Invitrogen, (Grand Island, NY) according to the manufacturer's protocol. Cells were harvested after 72 h for HO-1 assessment levels.

The human GSTHO-1 expression vector was constructed as follows: HO-1 was sub-cloned in a eukaryotic expression vector (pEBG) fused by its amino end to a GST tag to allow its subsequent affinity purification (pull-down). The plasmid (2 μg) was transfected in PC3 cells using polyethylenimine (PEI) reagent from SIGMA-Aldrich (UK). Transfected cells were treated or not with IL6 (10 ng/μl, 24 h). Cell extracts from the GSTHO-1 expressing cells and controls were then prepared for pull-down assays under conditions that minimized disruption of interactions (low salt concentration and detergents).

### Cell Adhesion

Cells were analyzed for adhesion to collagen-coated plates (Purecol™ INAMED Collagen). PC3, hemin treated PC3, PC3HO-1, and PC3pcDNA3 (stably and transiently transfected) cells were seeded into previously collagen-coated 24-well plates. Cells were allowed to adhere. After 30 min of incubation, unbound cells were washed away with PBS. Attached cells were fixed in 100% methanol for 10 min and stained with 0.1% (w/v) Crystal Violet (Sigma, UK) Cells attached to the collagen-matrix were counted by light microscopy. Cells were plated in duplicates and four fields of view from each well were counted. Three independent experiments were carried out.

### RNA isolation and RT-qPCR (reverse transcription quantitative PCR)

Total RNA was isolated with the RNeasy Mini Kit (Qiagen). cDNAs were synthesized with RevertAid™ Premium First Strand cDNA Synthesis Kit (Fermentas) and used for real-time PCR amplification with Taq DNA Polymerase (Invitrogen) in a DNA Engine Opticon (MJ Research). Each PCR was performed in duplicate and three biological independent experiments were performed. Primers were designed to amplify a 100 bp region present in the fully mature RNA species of HO-1 (5'-GAGTGTAAGGACCCATCGGA-3' and 5'-GCCAGCAACAAAGTGCAAG-3'); E- cadherin (5'-AAGGTTCACCCAGCACCTTGCA-3' and 5'-GGCAGAGGGACACACCAGTGTA-3'); β-catenin (5'-CATAACCTTTCCCATCATCGT-3' and 5'-TGTGGAGAGTTGTAATGGCA-3') and ACTB (5'- CGGTTGGCCTTAGGGTTCAGGGGGG-3' and 5'-GTGGGCCGCTCTAGGCACCA-3'). Data were analyzed by Opticon-3 software and normalized to β-actin and control. Errors were calculated as previously described [20].

### siRNA Transfection

siRNA was synthesized in 2'-deprotected, duplexed, de- salted, and purified form by Sigma (UK). The sense and antisense sequences of human HO-1 siRNA were as follows: sense, 5'-GGAGAUUGAGCGCAACAAGdTdT-3' and antisense 5'-CUUGUUGCGCUAAAUCUCCdTdT-3'). siRNA scramble (Dharmacon) was used as a negative control. PC3 cells were grown in 10 cm plates until 60% of confluence, treated with hemin (70 μM, 24 h), and then transfected using (PEI) reagent (SIGMA) in medium without FBS. After 5 h of incubation, 20% FBS/BRFF-HPC1 medium was added. Western Blot, RT-qPCR and immunofluorescence analyses were done 72 h post-transfection.

### Immunoblotting

Western blot assays were carried out as previously described [20]. Briefly, PCa cells were lysed with CelLytic M Cell Lysis Reagent (Sigma), incubated on ice for 20 min, centrifuged at 12,000 rpm for 3 min, and the supernatant was collected. Protein concentration was determined using the bicinchoninic acid (BCA) protein assay kit from SIGMA. Samples were then resolved by SDS-PAGE, transferred to a nitrocellulose membrane (Invitrogen,). Membranes were blocked for 1 h with 5% (w/v) non-fat milk in TBS-T (0.1% Tween-20 in 10 mM Tris-HCl pH 7.4), and then incubated with specific primary antibodies: anti-HO-1 (1:500) or anti-β-actin (1:40000) for 1 hr. After washes with TBS-T incubations with the appropriate secondary antibodies were performed. Specific protein bands were detected using ECL reagents (Amersham Ltd).

### Bioinformatics data analysis

The network was performed using GeneMANIA version 3.1.2 [22] (http://www.genemania.org/) with the down-regulated (≥2.0 fold) genes after hemin treatment or in the pcDNA3-HO-1 cells compared to the respective controls. Gene Ontology (GO) and KEGG pathway analysis was performed using the Database for Annotation, Visualization and Integrated Discovery (DAVID) v6.7 [23, 24] (http://david.abcc.ncifcrf.gov/). The HO-1 modulated (≥2.0 fold) genes were used as the test-list and the complete microarray gene list as the background-list. *P*≤0.05 was considered significant. Metacore from Thompson Reuters (version 6.15), was also used to perform the enrichment analysis of HO-1 modulated genes (http://thomsonreuters.com/metacore/).

### Immunofluorescence experiments

Cells were seeded in 12-well plate at a density of 1×10^5^ cells per well on coverslips overnight. Cells were treated with hemin as described above, fixed in ice-cold methanol, permeabilized for 10 min with 0.5% Triton X-100/PBS, washed twice with PBS and then blocked with 5% bovine serum albumin (BSA) in PBS. Cells were incubated overnight with primary antibodies diluted in 4% BSA/0.1% Tween-20 in PBS. Cells were washed with PBS and incubated with fluorescent secondary antibodies for 2 h. Negative controls were carried out using PBS instead of primary antibodies. Cells were washed, mounted in FluorSave Reagent (Merck Millipore), and imaged by confocal laser scanning microscopy with an Olympus Fluoview FV 1000 microscope (and its software). An Olympusnwater immersion 60x objective (1.20 N.A. UPLAN APO) objective was used and excitation and emission filter were as follows: Alexa-488: excitation, 488 nm; emission, band-pass (BP) 505-525 nm. Alexa-555: excitation, 543 nm; emission, BP 560-620 nm. Samples were imaged at room temperature.

Wide field microscopy was carried on using an Olympus IX71 microscope with an Olympus UApo water immersion 40x objective (1.15 N.A), a mercury arc lamp excitation, and suitable filters. Camera: Hamamatsu Orca CCD C4742-95. Samples were imaged at room temperature.

### Image processing for presentation

Confocal and wide field microscope images were processed for presentation with Image J (http://rsb.info.nih.gov, NIH). Background of each channel was subtracted and in some cases a median filter (radius: 1 pixel) was applied only for presentation.

### Quantitative microscopy and co-localization analysis

All the quantitative microscopy measurements were performed in individual cells (10-40 cells for each treatment or condition) as previously described [65]. For co-localization analysis Manders coefficients were calculated applying the Image J plugin intensity correlation analysis after background subtraction and individual cell segmentation as previously described [66].

### Intracellular distribution of E-cadherin, β-catenin, HO-1 and Muskelin

Intracellular distribution of different proteins was analyzed by a homemade routine created using Matlab. *Segmentation*. Channel backgrounds (median) were subtracted and segmentation was manually performed for each cell using the HO-1 (green) images. After cell segmentation, the nucleus was defined as the pixels were the DAPI signal (blue) was >60 cts. DIC and HO-1 images were used to define the plasma membrane region. This segmentation leaded to the generation of three masks: “cell”, “membrane” and “nucleus”. *Estimation of the cellular distribution of E-cadherin, β-catenin, HO-1, and Muskelin.* Fluorescence intensity values in the “membrane”, “nucleus” and in the “cell” were summed for the correspondent channel (E-cadherin, β-catenin, HO-1, Muskelin). Therefore, we obtained the total E-cadherin, β-catenin, HO-1, Muskelin and the plasma membrane or the nucleus values. To compute the distribution of the fluorescence, we calculated the ratio membrane/cell, nucleus/cell. This ‘internal calibration’ approach was chosen to remove the influence of the amplifier gain and the zoom factor for each image acquisition condition. *Calculation of cell perimeter.* Cell perimeter was measured using ImageJ software. Cells were manually segmented using DIC images and the perimeter was measured. *Calculation of the percentage of cellular contact (% cellular contact).* Using ImageJ we manually segmented each cell membrane and measured the “total perimeter”. Similarly, we segmented the contacted membrane and we measure its size (“shared perimeter”). With these two measurements we calculated the % cellular contact following equation 1.

$$\%\text{contact}=100\times \frac{\text{shared perimeter}}{\text{total perimeter}}$$ [equation 1]

### Pull-Down and Mass spectrometry analysis

Recombinant GSTHO-1 protein complexes were pull-downed with Glutathione-S-agarose coated beads and the pull-down fractions with the co-precipitated proteins were washed and loaded onto SDS-PAGE gels and stained with Colloidal Coomasie. The bands were excised, and proteins were reduced using dithiothreitol (DTT), alkylated with iodoacetamide and digested with trypsin. For MALDI-TOF/TOF analyses, peptides were desalted and concentrated in a C18 resin (Zip-Tips. Waters Coorporation). MS and MSMS spectra were acquired using *Ultraflex II* MALDI TOF/TOF (Bruker Daltonics). *Database analysis*: MALDI MS and MSMS spectra were analyzed using Flex Analysis and Biotools Software (Bruker Daltonics). The peaklists obtained were processed and compared with NCBI databases using Protein Prospector and/or Mascot Software. Searches were performed against the human genome, allowing a peptide tolerance of 200 ppm, and a fragment tolerance of 0.7 Da.To control for nonspecific binding, we compared HO-1GST-extracted proteins to those pulled down with GST. Only differential HO-1GST-binding proteins compared to GST-binding proteins were considered further.

### Human Prostate Cancer Xenograft Model

Mice xenografts using PC3HO-1 and PC3pcDNA3 cell lines were previously described [20].

### Statistical analysis

Results are shown as mean ± s.e.m of ‘*n*’ separate independent experiments unless otherwise is stated. Two tails Student's t-Test was used to ascertain statistical significance. *P*<0.05 (*) and *P*<0.01 (**) were considered significant.

### Supplemental information

Supplemental Information includes two supplemental tables for the gene ontology analysis of down-regulated genes in PC3HO-1 cells and hemin treated PC3 cells versus their respective control cell line and three 3 supplemental figures (Supplemental fig 1: bioinformatics enrichment ontology analysis using Metacore software (Thompson Reuters); Supplemental fig 2: Mascot search engine results; Supplemental fig 3: Muskelin immunofluorescence assays in PCa cells lines.

## SUPPLEMENTARY FIGURES AND TABLES




