# Supplementary material for: Heme-oxygenase-1 implications in cell morphology and the adhesive behavior of prostate cancer cells
Source: Oncotarget. 2014 Mar 16;5(12):4087–102. doi: 10.18632/oncotarget.1826 (PMC4147308; doi:10.18632/oncotarget.1826)
Supplement: Supplementary file 1 [file oncotarget-05-4087-s001.pdf]

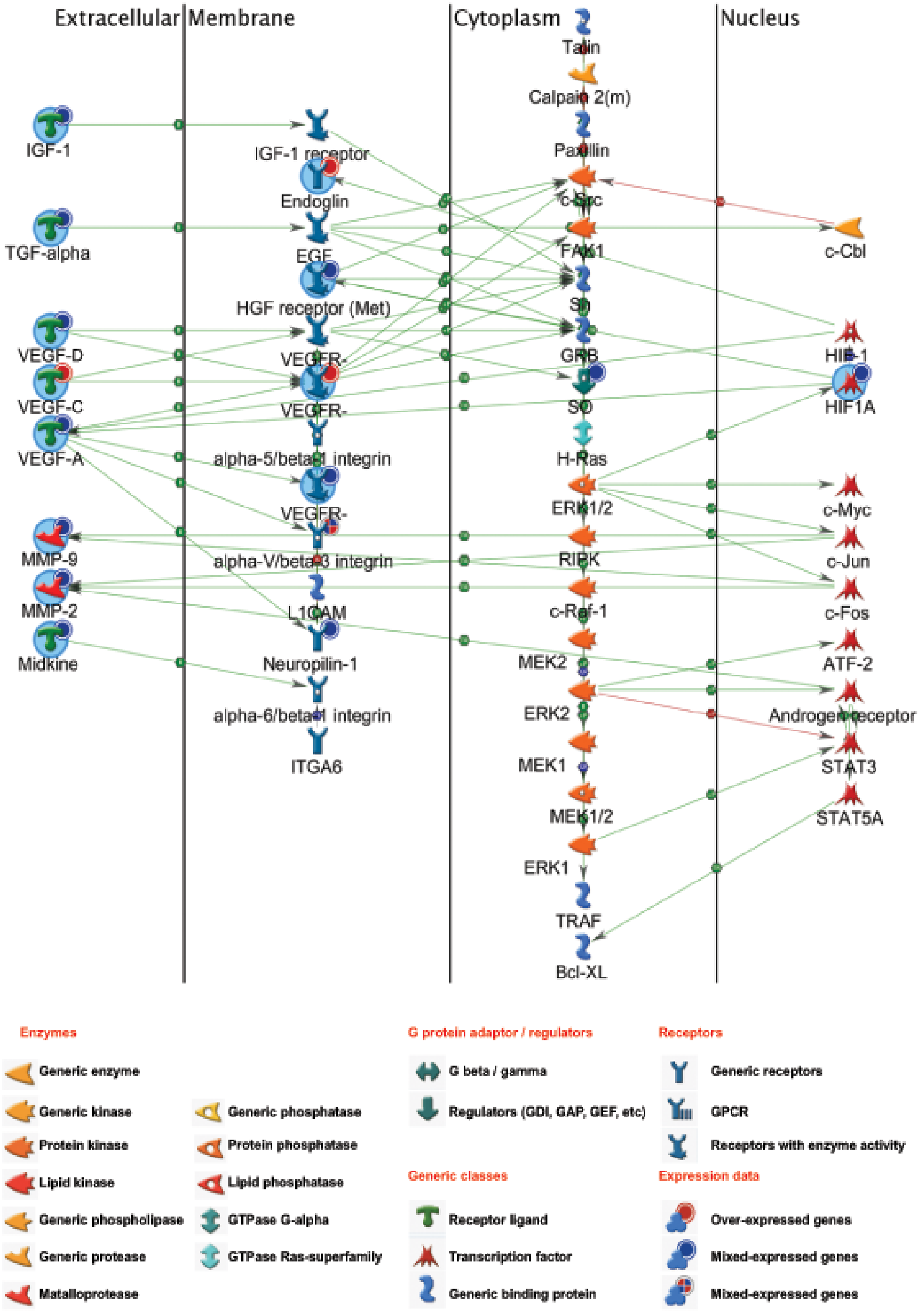

SUPPLEMENTAL FIGURE 1: A) Metacore software (Thompson Reuters) was used to perform the enrichment ontology analysis. The *disease (by biomarker)* ontology was performed. The enrichment profile between specific HO-1 modulated (≥2.0 fold) genes in the PC3HO-1 *vs.* control cells, showed a *P*-value of 2.767×10^{-19} for the Prostatic Epithelial Neoplasia Disorder

(PIN) associated networks. The layout shows the subcellular localization left to right of a sub-network enriched within this category with the seed nodes = HO-1 modulated genes, which were ranked by a *P*-value and G-Score. Light blue-circled genes represent HO-1 modulated genes. Connecting arrows (green-positive effect, red-negative effect) represent either binding, phosphorylation or transcription regulation.

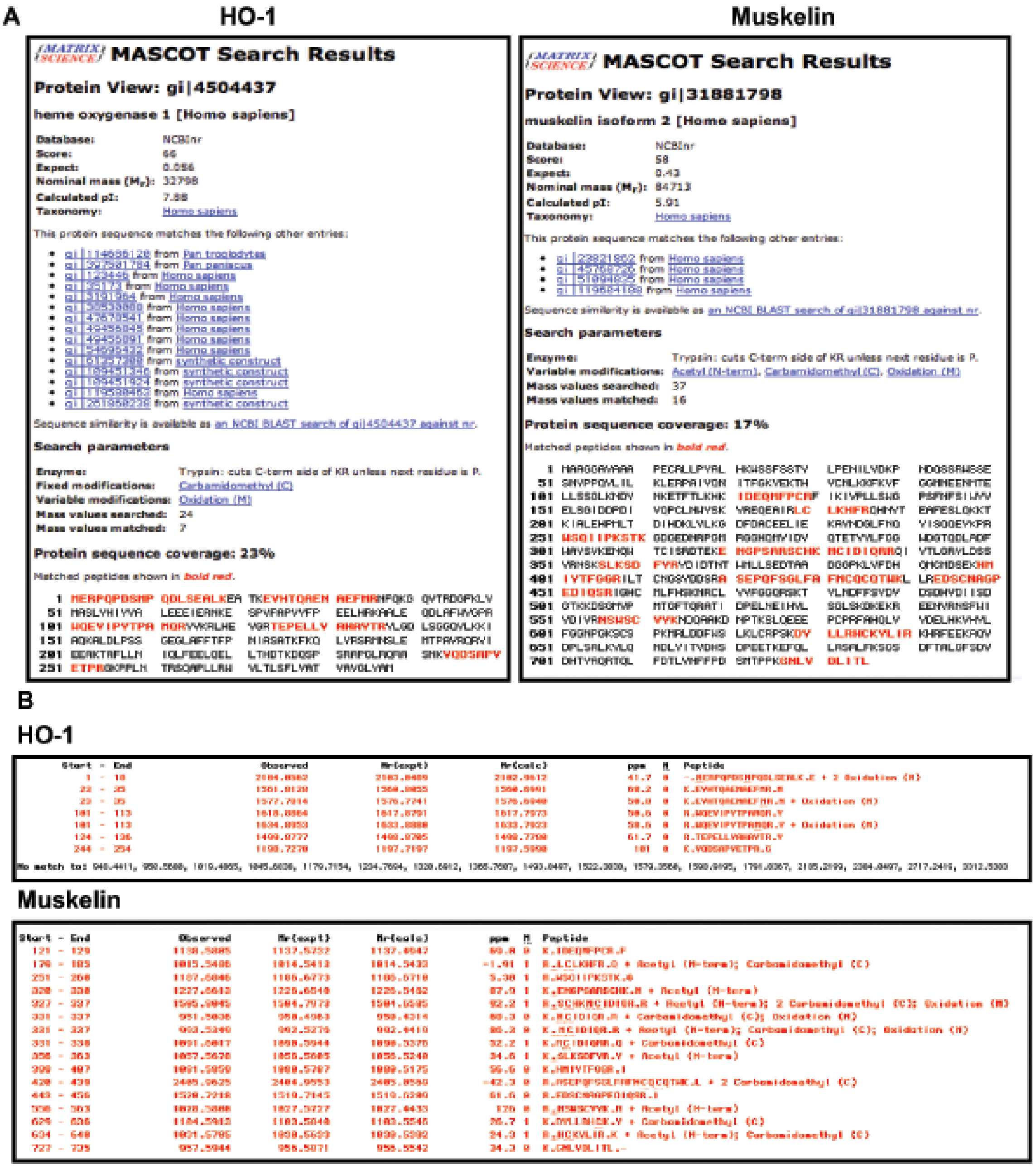

SUPPLEMENTAL FIGURE 2: A) Mascot search engine results. HO-1 sequence coverage can be observed. Peptide sequences highlighted in red indicate the theoretical masses that coincide with the experimental masses. Peptide detail with their modifications and statistical error are

shown in the lower panel. B) Mascot search engine results show Muskelin sequence coverage. Peptide sequences highlighted in red indicate the theoretical masses that coincide with the experimental masses. Peptide detail with their modifications and statistical error are shown in the lower panel.

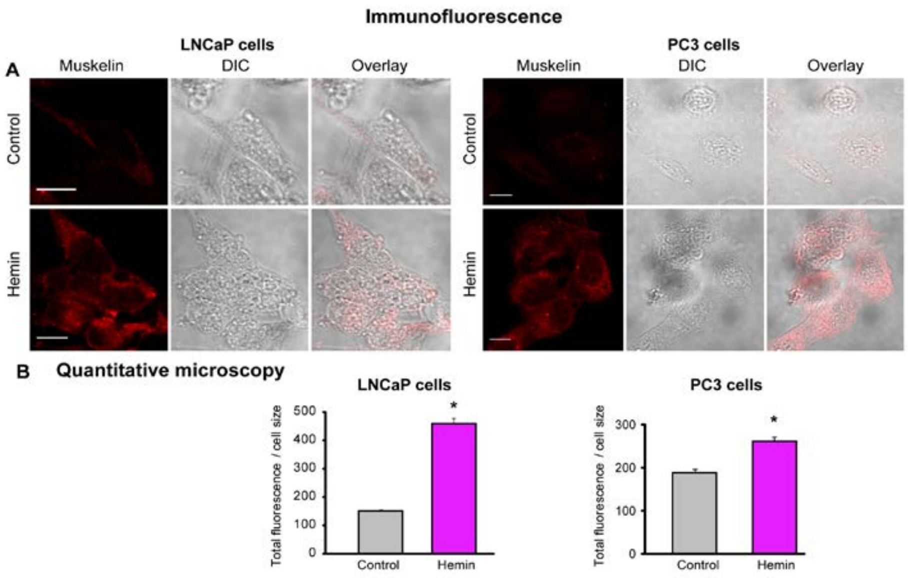

SUPPLEMENTAL FIGURE 3: A) Muskelin immunofluorescence assays in PC3 and LNCaP cells exposed to hemin (70 µM, 24 h) or vehicle (control). B) The florescence intensity for Muskelin normalized to the cell size was calculated for each experimental condition using Matlab (*P<0.01; n≥30 cells for each condition).

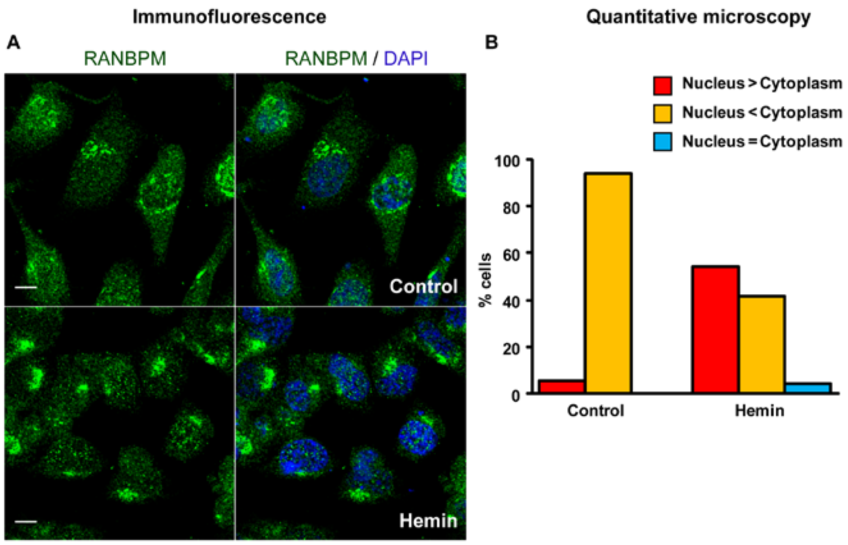

SUPPLEMENTAL FIGURE 4: A) RANBPM immunofluorescence assays in PC3 cells exposed to hemin (70 µM, 24 h) or vehicle (control). B) Subcellular localization of RANBPM in cells shown in A was quantified using Matlab by measuring the fluorescence intensity in the cytoplasm and within the nucleus (n=17 cells for vehicle and n=24 cells for hemin). Based on this measurement the cells were scored as follows: cells showing mostly cytoplasmic staining (total fluorescence nucleus < total fluorescence cytoplasm), cells showing mostly nuclear staining (total fluorescence nucleus > total fluorescence cytoplasm), and cells showing equal cytoplasmic and nuclear intensity. Scale bar, 10µM.
