# Supplementary material for: Heme-oxygenase-1 implications in cell morphology and the adhesive behavior of prostate cancer cells
Source: Oncotarget. 2014 Mar 16;5(12):4087–102. doi: 10.18632/oncotarget.1826 (PMC4147308; doi:10.18632/oncotarget.1826)
Supplement: Supplementary file 2 [file oncotarget-05-4087-s002.pdf]

Heme-oxygenase-1 implications in cell morphology and the adhesive behavior of prostate cancer cells – Gueron et al

Supplemental Table S1: Gene ontology analysis of down-regulated genes in hemin treated PC3 cells versus their respective control cell line.

Category	Term	p value	- log (p)	Genes
BP	G-protein coupled receptor protein signaling	3.5E-02	1.46	CXCL1, CXCL5, CXC
BP	Immune response	8.2E-02	1.08	CXCL1, CXCL5, CXCL3, VEGFA
MF	Cytokine activity	4.2E-02	1.37	CXCL1, CXCL5, CXCL3, VEGFA
MF	Chemokine activity	7.4E-02	1.13	CXCL1, CXCL5, CXCL3
MF	Chemokine receptor binding	7.4E-02	1.13	CXCL1, CXCL5, CXCL3
CC	Extracellular space	3.3E-02	1.48	CXCL1, CXCL5, CXCL3, MMP9, VEGFA, ANGPT2
KEGG path	Cytokine-cytokine receptor interaction	8.0E-04	3.09	CXCL1, CXCL5, CXCL3, VEGFA
KEGG path	Chemokine signaling	2.8E-03	2.56	CXCL1, CXCL5, CXCL3

Supplemental Table S2: Gene ontology analysis of down-regulated genes in PC3HO-1 cells versus their respective control cell line.

Category	Term	p value	- log (p)	Genes
BP	G-protein coupled receptor protein signaling	3.1E-03	2.51	CXCL1, IL8, CXCL5, CXCL3, CXCL10
BP	Locomotion regulation	3.4E-03	2.47	IL6, IL8, MMP9, VEGFA, ANGPTL3, THBS1, CXCL10
BP	Immune response	3.9E-03	2.41	CXCL1, IL6, IL8, CXCL5, CXCL3, VEGFA, THBS1, CXCL10
BP	Cell surface receptor linked signaling	8.1E-03	2.09	CXCL1, IL8, CXCL5, CXCL3, VEGFA, ANGPT1, ANGPTL3, FIGF, CXCL10
BP	Cell migration regulation	1.2E-02	1.91	IL6, MMP9, VEGFA, ANGPTL3, THBS1, CXCL10
BP	Locomotory behavior	1.5E-02	1.82	CXCL1, IL6, IL8, CXCL5, CXCL3, CXCL10
BP	Chemotaxis regulation	2.3E-02	1.63	IL6, IL8, VEGFA, THBS1
BP	Posttranscriptional regulation of gene expression	3.1E-02	1.50	IL6, VEGFA, THBS1
BP	Cell proliferation regulation	3.5E-02	1.45	CXCL1, IL6, IL8, CXCL5, VEGFA, THBS1, FIGF, CXCL10
MF	Cytokine activity	1.3E-02	1.87	CXCL1, IL6, IL8, CXCL5, CXCL3, VEGFA, CXCL10
MF	Chemokine activity	2.3E-02	1.64	CXCL1, IL8, CXCL5, CXCL3, CXCL10
MF	Chemokine receptor binding	2.3E-02	1.64	CXCL1, IL8, CXCL5, CXCL3, CXCL10
CC	Extracellular space	5.0E-04	3.31	CXCL1, IL6, IL8, CXCL5, CXCL3, MMP9, VEGFA, ANGPT1, ANGPTL3, THBS1, FIGF, CXCL10
CC	Platelet alpha granule lumen	6.9E-02	1.16	VEGFA, THBS1, FIGF
KEGG path	Cytokine-cytokine receptor interaction	3.3E-08	7.48	CXCL1, IL6, IL8, CXCL5, CXCL3, VEGFA, FIGF, CXCL10
KEGG path	Chemokine signaling	1.1E-05	4.95	CXCL1, IL8, CXCL5, CXCL3, CXCL10
KEGG path	Bladder cancer	5.0E-05	4.30	IL8, MMP9, VEGFA, THBS1, FIGF
KEGG path	Pathways in cancer	2.3E-04	3.65	IL6, IL8, MMP9, VEGFA, FIGF
KEGG path	NOD-like receptor signaling	6.5E-03	2.18	CXCL1, IL6, IL8
KEGG path	Toll-like receptor signaling pathway	1.1E-02	1.97	IL6, IL8, CXCL10
KEGG path	Focal adhesion	3.5E-02	1.44	VEGFA, THBS1, FIGF
